# Screening of New Microsatellite DNA Markers from the Genome of *Platyeriocheir formosa*

**DOI:** 10.3390/ijms13055598

**Published:** 2012-05-09

**Authors:** Hui-Ling Cheng, Yan-Horn Lee, Dai-Shion Hsiung, Mei-Chen Tseng

**Affiliations:** 1 Department of Environmental Resources Management, Chia Nan University of Pharmacy and Science, Tainan 717, Taiwan; E-Mail: lynn62359@mail.chna.edu.tw; 2 Biotechnology Division, Fisheries Research Institute Council of Agriculture, Tungkang, Pingtung 928, Taiwan; E-Mail: leeyh4331@hotmail.com; 3 Department of Aquaculture, National Pingtung University of Science and Technology, Pingtung 912, Taiwan; E-Mail: bear000000@yahoo.com.tw

**Keywords:** catadromous, crab, cross-specific amplification, polymorphic loci

## Abstract

The catadromous *Platyeriocheir formosa* is a crab endemic in Taiwan. To conserve *P. formosa* population diversity and ensure the sustainable use of this natural resource, we have developed new genetic markers, 17 polymorphic microsatellite loci, to promote the study of its population genetics in the future. In this study, more than 70 microsatellite sequences were found. Among these, 18 loci were selected to analyze the genetic diversity of *P. formosa*. With the exception of the Pfo15 locus, all of the remaining loci were polymorphic with allelic numbers ranging from 3–14. Heterozygosity within all 17 polymorphic loci ranged from 0.2–0.95 with an average of 0.55, which suggested that these loci are proper markers for studying population genetics. After we tested cross-specific amplification, eight and six primer sets could be successfully used for the amplification of microsatellite loci in morphologically similar *Eriocheir sinensis* and *E. japonica*, respectively; this suggests that they are useful markers for closely related species.

## 1. Introduction

*Platyeriocheir formosa*, the unique species in the genus, is an endemic Taiwanese species of crab which is mainly distributed in rivers of eastern Taiwan [1] and which has a peculiar life history of spawning in the sea and growing up in rivers. The reproductive period of *P. formosa* is during spring and summer which greatly differs from morphologically similar *Eriocheir* species that spawn in winter. Eggs hatch into zoea which leave the female individual and begin a planktonic life in the sea. After passing through five ecdysis cycles, they metamorphose into megalopa that become submersed in an estuary until a second metamorphosis into juvenile crabs occurs [2]. In the summer, juvenile crabs move into rivers where they grow until adulthood. As the reproductive gland gradually matures, adults move from their habitats in middle and upstream reaches of rivers to coastal waters for mating and reproduction. The genetic diversity of *P. formosa* was analyzed using mitochondrial DNA cytochrome oxidase subunit I (COI) sequences, and results suggest that the species maintains high genetic polymorphism [3]. However, there was an insignificant genetic structure between samples from different streams. The primary reason was inferred to be a result of gene flow of *P. formosa* as their zoeal and megalopa stages disperse to different estuaries by ocean current transport.

*Platyeriocheir formosa* is a highly economic aquatic resource in Taiwan. Because the complete artificial culture of *P. formosa* has not yet succeeded, people catch large numbers of *P. formosa* from wild habitats and sell them. The wild population has to tolerate this intense artificial exploitation pressure. Unfortunately, when typhoon Morakot hit Taiwan in 2009, many rivers suffered severely, including mudflows that covered riverbeds which resulted in a significant decrease of population abundances of organisms such as *P. formosa* in rivers. It is clear that conservation work on *P. formosa* should be taken seriously. Because microsatellites have large mutation rates of 10^−5^–10^−2^ per generation [4], they are widely used as markers for studying genetic mapping, population structure, kinship, evolutionary genetics, and genetic diseases [5–10]. For example, Xu and Liu developed microsatellite markers for the genetic analysis of the swimming crab, *Portunus trituberculatus* [11]. Microsatellite loci of the blue crab (*Callinectes sapidus*), the largest and most successful commercial and recreational fishery in Chesapeake Bay, USA [12], were cloned from the genome for forensic identification, pedigree analysis, and determination of the population structure [13].

To conserve *P. formosa* population diversity and ensure the sustainable use of this natural resource, the genetic structure and population dynamics must be determined. In this study, we developed new genetic markers, 17 polymorphic microsatellite loci, to promote the study of population genetics in the future.

## 2. Results and Discussion

Among about 2400 clones screened, 103 had positive signals. After sequencing, 97 different microsatellite sequences were determined including 70 GA/GT microsatellite sequences and 27 non-GA/GT components, e.g., TA, GC, and GGA repeats. Microsatellites were divided into three categories. For perfect microsatellites, the core region consists of tandem repeats. Tandem repeats interrupted by one or more additional or substituted nucleotides are called interrupted microsatellites. Compound repeated loci are composed of stretches of different repeated motifs [14]. In this study, all 70 cloned GA/GT loci included 49 (70%) perfect, 15 (21.4%) interrupted, and six (8.6%) compound microsatellite sequences, which suggested that the GA/GT microsatellites mainly consist of perfect repeat sequences in the genome of *P. formosa*. Total numbers of repeated motifs in the core region ranged from five to 45 for (GA)_n_ microsatellites and from five to 50 for (GT)_n_ microsatellites. Primers were designed for only 39 of the 70 GA/GT microsatellite sequences. The remaining microsatellites sequenced were not used due to the occurrence of a repeated site in one of the flanking regions, or because they were short. We tested various polymerase chain reaction (PCR) conditions with different concentrations of MgCl_2_, annealing temperatures, and numbers of PCR cycles. After resolution on 2% agarose gels, 20 loci did not produce specific amplifications or were an inaccurate size; this was probably due to unfit primer design. However, we succeeded in testing the amplification of 19 loci that yielded consistent PCR products corresponding to a single locus of the expected size. One of the 19 loci (Pfo11) was monomorphic in all individuals screened. The Pfo15 locus was homozygous in all individuals. The remaining 17 loci have heterozygous genotypes. Primer sequences and PCR conditions of these polymorphic 18 loci that were correctly amplified are given in Table 1. Allelic numbers within all 18 loci ranged from 3–14. The effective allelic number ranged from 2.25–10.26. Allelic sizes within these loci ranged from 68–239 bp. The allelic sizes of Pfo15, -31, and -34 were all shorter than 100 bp. When excluding the homozygous Pfo15 locus, the observed heterozygosity (*H*_O_) ranged from 0.20–0.95 with an average of 0.55. The expected heterozygosity (*H*_E_) ranged from 0.57–0.93 with an average of 0.818. Thirteen of these loci exhibited departure from Hardy-Weinberg (HW) equilibrium, which may suggest that *P. formosa* has a small effective population size, suffers an intense inbreeding effect or null allele effect or other possibilities. Burrow’s composite measure for linkage disequilibrium (LD) among the 18 loci was estimated for the entire dataset. The total variance of interlocus allelic disequilibrium (*D*_IT_^2^ = 0.018) exhibited slight disequilibrium.

In contrast, a higher mean heterozygosity (*H*_O_ > 0.8) was found in the marine swimming crab, *Portunus trituberculatus* [11], while a similar value of 0.55 was found in the catadromous crab, *E. sinensis* [15]. The mean allelic number per locus (*na*) in the catadromous *P. formosa* was estimated to be 9.61, which was lower than that of the marine *Po. trituberculatus* (*na* = 22), but higher than that of *E. sinensis* on average (*na* = 4.94) [11,15]. These results agree with the observation that catadromous *P. formosa* have lower mean allelic numbers and genetic diversity than marine *Po. trituberculatus* species. Previous studies of microsatellite primers showed that they may be useful across a wide taxonomic range [16,17]. In this study, we also proved their cross-specific application as shown in Table 1. From these results, eight and six of the 18 microsatellite loci were successfully cross-amplified in *E. sinensis* and *E. japonica*, respectively. In the long-term pursuit of excellence in microsatellite studies of *P. formosa*, we provide a preliminary description of the characteristics of 18 novel microsatellite loci and their advantages as markers to study genetic diversity and population dynamics.

## 3. Experimental Section

### 3.1. Sample Collection

In total, 40 *Platyeriocheir formosa* specimens were collected in November 2010 from Jin-Luen, Taitung (120°55′ E, 22°32′ N), southeastern Taiwan. The morphologically similar river crab, *Eriocheir japonica*, was collected from the Houlong River, in central Taiwan. Another economic exotic species of river crab, *E. sinensis*, was bought from an aquaculture farm in Pingtung, southern Taiwan.

### 3.2. Genomic DNA Isolation

Muscle tissues from all specimens were preserved in 95% ethanol until DNA extraction. To obtain a large quantity of DNA for microsatellite library preparation, genomic DNA was isolated and purified from muscle tissues of one individual. Five hundred milligrams of tissues with 1 mL lysis buffer was digested with 55 μL proteinase K solution—10 mM Tris-HCl (pH 8.0), 2 mM ethylenediaminetetra-acetic acid (EDTA), 10 mM NaCl, 1% sodium dodecylsulfate (SDS), 10 mg/mL dithiothreitol (DTT), and 0.5 mg/mL proteinase K. DNA extraction was carried out using the method described in reference [18]. To quickly and conveniently purify small amounts of DNA from all specimens for the polymerase chain reaction (PCR), a Puregene core kit A (Qiagen, Valencia, CA, USA) was used in this study.

### 3.3. Screening of GA/GT Microsatellite Sequences

High-molecular-weight DNA from the genome DNA of one individual was digested with the *Alu*I, *Hae*III, and *Rsa*I restriction enzymes (BioLabs, Ipswich, MA, USA) according to the manufacturer’s instructions. Fragments of 200–800 nt were gel-purified using a GeneMark DNA Clean/Extraction Kit (Genemark Technology, Tainan, Taiwan) and inserted into the *Sma*I blunt site of pUC 18 (BioLabs). Recombinants carrying the fragments were transformed to a competent *Escherichia coli* DH5α strain. The library was plated onto 2YT medium plates, and colonies were lifted onto Whatman filters. These plates were placed in an incubator until the colonies were restored. All filters were immersed in freshly prepared denaturing solution (0.5 M NaOH and 1.5 M NaCl) for 7 min, twice in neutralizing solution (0.5 M Tris-HCl and 1.5 M NaCl, at pH 8.0) for 3 min, and in 2–4× SSC for 4 min. They were then dried in an 80 °C oven for 1–2 h and preserved at −20 °C until hybridization. Before prehybridization, we washed these filters using a solution of 2× SSC and 0.2% SDS. Prehybridization proceeded with hybridization buffer [19] for 1 h at 60 °C. The probes used for hybridization were biotinylated oligo DNA (GT)_10_ and (GA)_10_. Hybridization was carried out overnight at 60 °C using a hybridization oven.

After hybridization, filters were washed twice in primary washing buffer (1× SSC and 0.2% SDS) at room temperature, washed once in secondary washing buffer (0.5× SSC and 0.2% SDS) at room temperature, and washed twice in 0.1× SSC and 0.2% SDS at 60 °C. These filters were immersed in blocking solution (Amersham Biosciences, Piscataway, NJ, USA) for 30 min and then washed three times in TBS buffer (0.05 M Tris-HCl and 0.15 M NaCl, at pH 7.5) for 10 min. The filters were incubated in 10 mL TBS buffer with 2 μL streptavidin-alkaline phosphatase (1 mg/mL) and 0.05% Tween 20 and then washed three times in TBS buffer for 10 min each. Deep-blue colonies were visualized by immersion in an NBT/BCIP solution for 1 h. Positive clones were sequenced on an Applied Biosystems automated DNA sequencer 377 vers. 3.3 (ABI, Foster City, CA, USA) using a Bigdye sequencing kit (PE Applied Biosystems, Foster City, CA, USA).

### 3.4. Genotyping and Analysis

Paired primers of 15–21 nt long were designed using DNASTAR Primer Select software (vers. 4.0) (DNASTAR, Madison, WI, USA) for the 39 clones containing perfect repeat sequences. A PCR was performed in a volume of 25 μL including ~10 ng genomic DNA, 10 pmol of the reverse primer, 10 pmol of the forward primer, 25 mM dNTP, 0.05–0.1 mM MgCl_2_, 10× buffer, and 0.5 U *Taq* polymerase (Takara Shuzo, Tokyo, Japan) with Milli-Q water. The PCR products were subjected to a 1.5% agarose gel, and allelic sizes were checked by comparison with a DNA ladder and the length of the original sequence. Forward primers were labeled with FAM, TAMRA, and HEX fluorescence markers. PCR amplifications were carried out in a Px2 Thermal Cycler (Thermo Fisher Scientific, Waltham, MA, USA) with the following temperature profile: 1 cycle of 95 °C for 4 min, followed by 38 cycles of 94 °C for 30 s and annealing at 52–60 °C for 30 s and 72 °C for 30 s. Each 5 μL of PCR product from three loci labeled with the different fluorescence tags was mixed and precipitated with 95% alcohol. Semiautomated genotyping was performed using a capillary ABI 3730*XL* DNA Analyzer (ABI). Genotypes were scored with GeneMapper 4.0 (ABI).

The total number of alleles (*na*) and effective allelic numbers were estimated for each locus using the program, Popgene [20]. Observed (*H*_O_) and expected (*H*_E_) heterozygosities were independently calculated for each locus [21]. Deviations from Hardy-Weinberg expectations (HWEs) were examined by an exact test using GENEPOP [22]. Linkage disequilibrium among all pairs of loci was determined using Burrow’s composite measure and χ^2^ values [23].

## 4. Conclusions

In conclusion, 18 out of 39 primer sets can successfully amplify microsatellite loci in *P. formosa*. The polymorphism of genetic variations suggested that these loci are proper genetic markers for addressing questions of population genetics and population dynamics of *P. formosa* in the future. In addition, we also tested the close related species and found that there are eight and six primer sets which can be used successfully for *E. sinensis* and *E. japonica*, respectively.

## Figures and Tables

**Table 1:** Primer sequences, characterization of the core region, and levels of genetic variation at 18 microsatellite loci from *Platyeriocheir formosa*.

Locus	Fluorescence labeling	Primer sequence (5′→3′)	Major repeats	*T*_a_ (°C)	Allelic size range (bp)	*na*	*ne*	*H*_O_/*H*_E_	*Cross species amplified For*		EMBL Accession no.
									*Eriocheir sinensis*	*E. japonica*	
Pfo 4	FAM	**F:** TGTGAGACGGCGGTTACGAG **R:** GAGCACTCTCCCTGGTCTTC	(CA)_29_	56	145–183	13	6.45	0.75/0.87	−	+	JQ582816
Pfo 5	HEX	**F:** TGTCCAACCGCTTTCTTTC **R:** CGAAGATAACAGTAATACGG	(TC)_6_	54	141–149	3	2.25	0.55/0.57	−	+	JQ582817
Pfo 7	HEX	**F:** ACTAATCCAATGCCTGCC **R:** CTATGCAGTCTCCTTCCGTAG	(GT)_22_	52	217–239	10	6.06	0.65/0.86	+	+	JQ582818
Pfo 9	TAMRA	**F:**TCTAGGCTGCAGCTTCATAG **R:**GGACGCATTAGCATAACA	(CA)_31_	54	156–194	14	9.88	0.45 */0.92	+	+	JQ582819
Pfo10	HEX	**F:** TACCACGTCCGTTCTAG **R:** CGTATAGGAGATTACTGG	(CA)_10_	50	94–128	8	7.27	0.65/0.88	−	−	JQ582820
Pfo12	TAMRA	**F:** TTCCTATCGCTCTCATCAGC **R:** TTGTCCAGTTCATACTG	(CA)_32_	56	186–216	14	7.84	0.45 */0.89	+	−	JQ582821
Pfo15	TAMRA	**F:** GAAGAGTGTGGCGGAG **R:** CTTGACACCTCGTGAGG	(GT)_16_	60	68–76	5	2.99	0.00 */0.68	+	−	JQ582822
Pfo18	FAM	**F:** GGAAGTGGTGGTAAAG **R:** CACTTAACAGGTGGACA	(CA)_33_	60	134–170	13	6.84	0.35 */0.88	+	−	JQ582823
Pfo19	FAM	**F:** GGTGATCTTGGGCACCG **R:** GATAGATACTTGAACACG	(CA)_32_	50	141–175	13	9.20	0.25 */0.91	−	−	JQ582824
Pfo31	FAM	**F:** GCACCACAGCGCTCTCTTAC **R:** GGTAGGAAGACAGTGCG	(CA)_17_	52	79–93	6	2.74	0.35 */0.65	−	+	JQ582825
Pfo34	TAMRA	**F:** GTAGAACTGACAGCC **R:** CTGGTGCCTTACCTGTC	(CA)_20_	50	71–77	3	2.38	0.70 */0.59	−	−	JQ582826
Pfo36	FAM	**F:** CTC GTTACTACACTCC **R:** CCATTCATATGCCATG	(CA)_35_	54	101–125	10	6.11	0.50 */0.86	−	−	JQ582827
Pfo37	TAMRA	**F:** AAGCTGGCTGACACCTG **R:** CGTCACCTTGCAATC	(CA)_31_	50	166–200	13	8.79	0.90/0.91	+	−	JQ582828
Pfo51	FAM	**F:** GTGCTCTGCGAAACG **R:** GGAGTGCTGGAGTAG	(CA)_18_	58	85–101	8	2.94	0.20 */0.68	−	−	JQ582829
Pfo52	HEX	**F:** GTATGTTGATGGCGTG **R:** AGAGTGTGGCGGAGGC	(CA)_12_	50	97–109	6	4.10	0.95*/0.78	−	−	JQ582830
Pfo54	TAMRA	**F:** GCATGCAAGAGCGTAG **R:** TGACAGACAGACTCC	(GT)_18_	50	141–173	10	5.52	0.70 */0.84	+	−	JQ582831
Pfo60	HEX	**F:** ACTATCACTAGGCTCAT **R:** TCAGTCTCGTATTCTC	(GT)_17_	50	118–146	11	7.62	0.55 */0.89	+	+	JQ582832
Pfo79	FAM	**F:** TTATCCTGATCCTGAG **R:** GACATAGCAGCAATAC	(GT)_26_	52	113–143	13	10.26	0.45 */0.93	−	−	JQ582833

*T*_a_, polymerase chain reaction (PCR) annealing temperature; *na*, observed number of alleles detected at each locus; *ne*, effective number of alleles; *H*_O_, observed heterozygosity within a sample; *H*_E_, expected heterozygosity within a sample.

* significant Hardy-Weinberg deviation (*p* < 0.05).
